# 

**Published:** 1897-03

**Authors:** 


					﻿Southern Medical Record.
A Monthly Journal of Medicine and Surgery.
Vol. XXVII.
ATLANTA, GA., MARCH, 1897.
No. 3
BERNARD WOLFF, M. D., Editor.
Associate Editors :
A. W. GRIGGS, M. D.	WILLIS F. WESTMORELAND, M. D.
j. McFadden gaston, m. d.
E. Y. CLARKE, General Manager.	B. M, ZETTLER, Business Manager.
TERMS: $1.00 Per Annum; Single Copy, 10 Cents.	Contents on Page 6.
Entered at the Post-Office at Atlanta, Ga., as Second-Class Matter.
The Foote & Davies Co., Atlanta, Ga.
				

